# Simultaneous and Efficient Production of Furfural and Subsequent Glucose in MTHF/H_2_O Biphasic System via Parameter Regulation

**DOI:** 10.3390/polym12030557

**Published:** 2020-03-03

**Authors:** Shaolong Sun, Xuefei Cao, Huiling Li, Yingbo Zhu, Yijing Li, Wei Jiang, Yang Wang, Shaoni Sun

**Affiliations:** 1 College of Natural Resources and Environment, South China Agricultural University, Guangzhou 510642, China; wyscyz2015@163.com; 2 Beijing Key Laboratory of Lignocellulosic Chemistry, Beijing Forestry University, Beijing 100083, China; caoxuefei@bjfu.edu.cn (X.C.); zhuyingbo527@163.com (Y.Z.); lyjing18@163.com (Y.L.); 3 State Key Laboratory of Bio-Fibers and Eco-Textiles, Qingdao University, Qingdao 266071, China; weijiangqd@qdu.edu.cn; 4 College of Forestry and Landscape Architecture, South China Agricultural University, Guangzhou 510642, China; lihl@scau.edu.cn

**Keywords:** MTHF/H_2_O, biphasic system, furfural, enzymatic hydrolysis, cornstalk

## Abstract

Efficient production of furfural from cornstalk in 2-Methyltetrahydrofuran/aqueous (MTHF/H_2_O) biphasic system via parameter regulation (e.g., V_MTHF_/V_H2O_, temperature, time, and H_2_SO_4_ concentration) was proposed. The resulting solid residues achieved from the different MTHF/H_2_O system conditions for furfural production were also to prepare glucose by adding cellulases to increase the high-value applications of cornstalk. A maximum furfural yield (68.1%) was obtained based on reaction condition (V_MTHF_:V_H2O_ = 1:1, 170 °C, 60 min, 0.05 M H_2_SO_4_). Among these parameters, the concentration of H_2_SO_4_ had the most obvious effect on the furfural production. The glucose yields of the residues acquired from different MTHF/H_2_O processes were enhanced and then a maximum value of 78.9% based on the maximum furfural production conditions was observed. Single factor may not be sufficient to detail the difference in glucose production, and several factors affected the hydrolysis efficiency of the residues. Overall, the MTHF/H_2_O system effectively converted cornstalk into furfural and glucose via a simple and environment-friendly process, thus was an ideal manner for the food industries.

## 1. Introduction

Furfural is a high value platform compound that has potential to produce bio-based chemical in the many industries and interests amongst scientists that surpasses [1,2,3]. Generally, agricultural and forestry biomasses are some of the main raw materials for furfural production because they contain rich hemicellulosic polymer constituents [4]. Cornstalk is often used for mulching. In fact, cornstalk is an ideal source for furfural production because it is xylan-rich polymers. Unfortunately, cornstalk is intractable for disintegrating hemicelluloses owing to the rigid and dense cell wall structure [5]. Therefore, a pretreatment process is usually required to destroy the inherent structure of cornstalk, thereby improving the furfural production and then glucose via enzymatic hydrolysis [6].

To date, diverse physical, chemical, and integrated processes have been applied to reduce recalcitrance and improve furfural preparation for different lignocelluloses, including the hydrothermal treatment, steam distillation, organosolv treatment, ionic liquid, and biphasic system [7,8,9,10,11,12]. Generally, furfural production from hemicellulosic polymer constituent of lignocelluloses during the pretreatment must undergo depolymerization of hemicelluloses and then dehydration of D-xylose reactions under acidic conditions [13]. Initially, furfural was prepared using a high concentration of acid solution. However, this method was liable to cause damage to the equipment, and the waste liquid produced caused environmental pollution. In addition, the furfural was also prepared by a single aqueous system under various mild acid conditions. However, due to the limited solubility of furfural in single aqueous system and the lack of effective prevention of side reactions, the furfural yield was very low [14]. Thus, a potential pathway with organic/aqueous biphasic system for enhancing the furfural yield was proposed since its high efficiency and mild treatment process [12]. It is worth noting that the furfural component prepared is rapidly transferred from aqueous phase to organic phase during the biphasic system, preventing the occurrence of side reactions in time [15]. Generally, alkylphenol, *γ*-valerolactone, toluene, methyl isobutyl ketone (MIBK), and 2-methyltetrahydrofuran (MTHF) were proposed in the biphasic system process as an organic phase [16,17,18,19,20]. Among these solvents, MTHF merits wide attention due to its relatively low-cost, easy recovery, and excellent stability in acidic conditions [21]. Hence, in-depth systematic research on furfural production from cornstalk by the MTHF/H_2_O biphasic system process was very necessary. In the previous studies, the use of the MTHF/H_2_O process was mainly focused on the preparation of furfural from different biomasses [22,23,24]. In the MTHF/H_2_O system, during the degradation of biomasses, the hemicellulose component is firstly degraded to furfural in the aqueous phase, and then transferred to MTHF, which prevents its further decomposition in order to obtain a high furfural yield. However, as a biorefinery process, the resulting cellulose-rich solid residue collected from the MTHF/H_2_O process should receive particular concern due to its high value in developing biobased materials and chemicals [25]. For glucose yield of resulting cellulose residue by enzymatic hydrolysis in biomasses, many factors affect its hydrolysis ratio, such as content and structure of lignin and hemicelluloses, surface morphology, crystallinity as well as polymerization degree of cellulose [26,27]. Therefore, the subsequent residue used to produce glucose by adding cellulases should be systematically analyzed in the current process. The information will help develop renewable, usable, and cellulose-based value-added products, such as bioethanol.

In the present study, efficient production of furfural from cornstalk in MTHF/H_2_O biphasic process via parameter regulation (e.g., V_MTHF_/V_H2O_, temperature, time and H_2_SO_4_ concentration) was proposed and analyzed by a high-performance liquid chromatography (HPLC). In addition, the resulting residues achieved from the different MTHF/H_2_O system processes were also to produce glucose by adding cellulases. A variety of physical and chemical characteristics of the resulting residues collected were also analyzed. Based on the above experiments and analysis, all data will help to understand the high value utilization of the polysaccharide components of cornstalk by the MTHF/H_2_O biphasic process.

## 2. Materials and Methods

### 2.1. Materials

Cornstalk was received from Hebei province, China. The cornstalk was ground to 40–60 mesh by using a micro plant grinding machine, and removed extractives by using toluene/ethanol (2:1, v/v), then dried under the conditions of 60 °C for 12 h as raw material (RM). The chemical compositions of RM (%, w/w) were measured to be cellulose 38.7% (glucan) and hemicelluloses 25.1% (xylan, arabinan, and galactan) and lignin 21.6% (acid insoluble lignin 19.8% and acid soluble lignin 1.8%), according to National Renewable Energy Laboratory’s (NREL) standard analytical procedure [28]. MTHF was purchased from Sigma-Aldrich. Enzyme (Cellic@ CTec2, 100 FPU/mL) was obtained by Novozymes (Beijing, China).

### 2.2. Preparation of Furfural by the MTHF/H_2_O System

Furfural production from cornstalk by the MTHF/H_2_O biphasic process was conducted in a batch reactor made of hastelloy C-276. In a typical reaction, 3 g RM and 60 mL MTHF/H_2_O solution with 0–0.1 M H_2_SO_4_ were added into a 100 mL reactor at 500 rpm under magnetic stirring conditions, in which the volume ratios of MTHF/H_2_O solution (V_MTHF_:V_H2O_) were set to 3:1, 2:1, 1:1 and 1:2, respectively. The reactor with RM was heated to 140–190 °C for 15–120 min. After the process finished, the reactor was cooled to room temperature, and then the liquor fractions and cellulose-rich solid residues were separated by a Buchner funnel. The concentrations of furfural in liquor fractions were measured. The specific determination process was described in a previously published article [19]. The residues were washed with deionized water and then dried at 80 °C for 12 h and then labeled as R_X-Y-Z_. X, Y, and Z were corresponding to temperature, time, and H_2_SO_4_ concentration of the MTHF/H_2_O system, respectively.

### 2.3. Enzymatic Hydrolysis

The cellulose-rich residues collected from the different MTHF/H_2_O processes were hydrolyzed by adding cellulases to prepare glucose. The hydrolysis experiments were carried out at 50 °C for 60 h. The specific hydrolysis conditions and the determination of the glucose sugar were detailed in the previous article in a recent publication [19].

### 2.4. Analysis Methods

The chemical compositions (%, w/w) of the resulting residues collected via different MTHF/H_2_O system processes were also assessed by the NREL method [28]. SEM images of RM and resulting residues were recorded by a JSM 6700F NT (Tokyo, Japan). FT-IR of RM and resulting residues were carried out using a FT-IR microscope (Thermo Nicolet Corporation, Madison, WI, USA) equipped. XRD of RM and resulting residues were performed via a D/MAX 2500PC diffractometer (Rigaku Corporation, Tokyo, Japan). The crystallinity indexes (CrIs) of the RM and resulting residues were measured from the ratio of the crystalline area to the total area of crystalline and amorphous peaks [19].

## 3. Results and Discussion

### 3.1. Furfural Yields by the MTHF/H_2_O System

**Figure 1:**
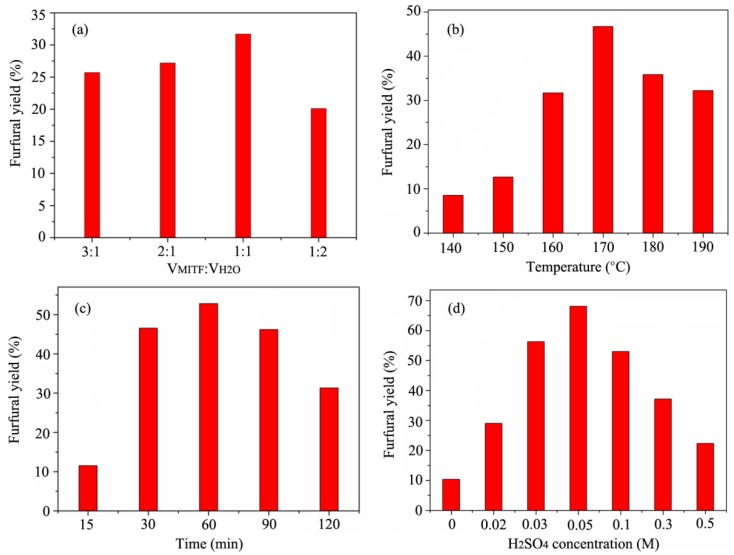
Furfural yields by the 2-Methyltetrahydrofuran/aqueous (MTHF/H_2_O) system under different conditions: (**a**) volume ratios of MTHF/H_2_O; (**b**) reaction temperatures; (**c**) reaction times; (**d**) H_2_SO_4_ concentrations.

The MTHF/H_2_O system process was proposed to reduce recalcitrance of cornstalk and enhance furfural yield. Figure 1 shows the effects of the volume ratios of MTHF/H_2_O, temperatures, times, and H_2_SO_4_ concentrations on the furfural yields from cornstalk. The MTHF/H_2_O process conditions greatly influenced the furfural yields. It can be seen from Figure 1a that the furfural yields were closely related to the volume ratios of MTHF/H_2_O. Under the reactions at 160 °C for 30 min with 0.1 M H_2_SO_4_ conditions, the furfural yields appeared gradual and was a slight enhancement from 25.7% to 31.7% with the volume ratios of MTHF/H_2_O reduced from 3:1 to 1:1. A possible reason was that the strengthening of xylan hemicelluloses depolymerization and D-xylose dehydration [19]. However, as the volume ratios of MTHF/H_2_O further reduced to 1:2, the furfural yields quickly reduced from 31.7% to 19.8%, which was mainly the furfural degradation during the MTHF/H_2_O process. This was also in keeping with the previous facts based on a MIBK/H_2_O biphasic system process [19]. In other words, the furfural constituent was labile and degraded quickly at low volume ratios of MTHF and H_2_O under the conditions given. Based on the results of Figure 1a, the volume ratios of MTHF and H_2_O of 1:1 were selected as a maximum production volume ratio of MTHF/H_2_O for furfural preparation from cornstalk.

Figure 1b exhibits the furfural yields at volume ratios of MTHF/H_2_O of 1:1 for 30 min with 0.1 M H_2_SO_4_ under different MTHF/H_2_O process temperatures (140–190 °C). It was found that the process temperature affected the furfural yield, releasing furfural from MTHF/H_2_O system with the highest yield (46.7%) acquired at 170 °C. In addition, the effect of the process times (15, 30, 60, 90, and 120 min) on furfural preparation was determined and the data are displayed in Figure 1c. The results demonstrated that the highest furfural yield (52.8%) was gained at 170 °C for 60 min with 0.1 M H_2_SO_4_. According to the report, whether in a single-phase aqueous solution or in an organic/aqueous biphasic system, the H_2_SO_4_ concentration significantly influenced the conversion of xylose to furfural [2,29]. Thus, under the conditions above (V_MTHF_:V_H2O_ = 1:1, 170 °C, 60 min), various H_2_SO_4_ concentrations (0, 0.02, 0.03, 0.05, 0.1, 0.3, and 0.5 M) were also added to the MTHF/H_2_O biphasic system for evaluating its impacted on the furfural production (Figure 1d). The furfural yield was obviously increased to 68.1% with 0.05 M H_2_SO_4_ concentration as compared to without adding H_2_SO_4_ (10.4%). However, when the H_2_SO_4_ concentrations further increased to 0.1–0.5 M, the furfural yields began to go down gradually, which was probably related to the further degradation of furfural under high concentration acid conditions. Therefore, it is not necessary to add high H_2_SO_4_ concentrations for furfural production during the MTHF/H_2_O system process. Based on the above analysis, it can be seen that the H_2_SO_4_ concentration was the most important parameter for furfural production because it had the most obvious influence on the furfural yield. In addition, during the MIBK/H_2_O process, the maximal furfural yield was 65.9% and the recovery of residue was 46.9% under an optimal reaction condition [19]. In the current study, a maximum production conditions with yield of 68.1% for furfural from cornstalk by MTHF/H_2_O process was achieved at 170 °C for 60 min with 0.05 M H_2_SO_4_ when the volume ratio of MTHF/H_2_O was 1:1. The furfural yield with MTHF/H_2_O process was higher than that of the MIBK/H_2_O process; thus, the MTHF/H_2_O process was a potential method to prepare furfural from cornstalk.

### 3.2. Yields and Constituent Analysis of the Resulting Residues

During the MTHF/H_2_O process, xylan hemicellulosic constituents were mainly converted into furfural via continuous depolymerization and dehydration processes [23]. Thus, yields and constituent analysis of the resulting residues changed significantly under the biphasic system conditions given. Table 1 shows the yields and chemical constituents of the resulting residues obtained by different MTHF/H_2_O processes. As compared to the residue yield (59.0%) at 170 °C for 60 min without adding H_2_SO_4_, the residue yields were continuously reduced (48.9–57.9%) with H_2_SO_4_ concentrations increased under the same temperature and time conditions. This was mainly due to the degradation of polysaccharides in cornstalk under the given conditions. In addition, as the MTHF/H_2_O process temperatures and times increased, the residue yields were also reduced.

As shown in Table 1, only a small amount of hemicellulosic polysaccharide (7.6%) was detected in R170-60-0, suggesting that the hemicellulosic constituents were prominently destroyed and degraded during the MTHF/H_2_O processes under the conditions given. The cellulose content of the residues first increase from 54.7% (in the experiment condition at 170 °C for 60 min without adding H_2_SO_4_) to 64.3% (in the experiment condition at 170 °C for 60 min with 0.05 M H_2_SO_4_), but then decreased to 51.1% at 170 °C for 60 min with 0.1 M H_2_SO_4_, suggesting that the degradation of partial cellulose occurred when the H_2_SO_4_ concentrations was higher than 0.05 M. Meanwhile, the difference in cellulose content of the residues presented a similar result with the MTHF/H_2_O process temperature elevated. In short, the MTHF/H_2_O process was effective to produce furfural and collect the cellulose-rich residues for the preparation of glucose by the hydrolysis experiment.

### 3.3. Enzymatic Hydrolysis of the RM and Resulting Residues

**Figure 2:**
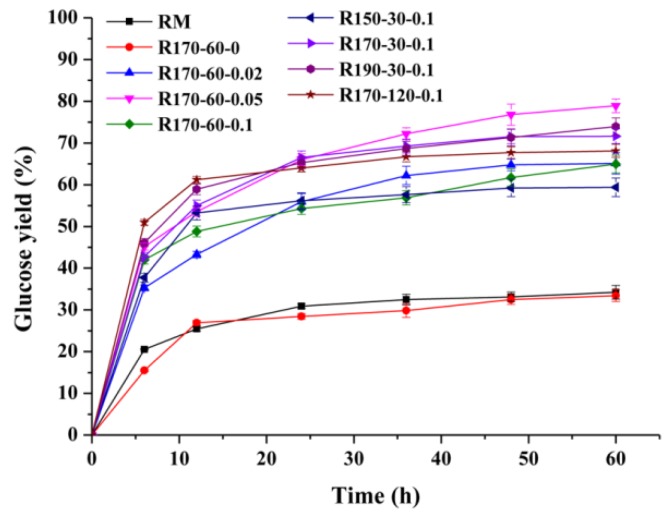
The glucose yields of the raw material and resulting solid residues by enzymatic hydrolysis.

The MTHF/H_2_O system not only enabled efficient preparation of furfural, but also simultaneously obtained cellulose-rich residue to prepare glucose by the hydrolysis experiment. In the current study, the enzymatic hydrolysis time of the substrate was set to 60 h. The glucose yields of the RM and resulting residues are emerged in Figure 2. As can be seen, the RM showed a low glucose yield (34.2%) because the dense structure hindered the accessibility of cellulases [19]. However, glucose yields of the resulting residues displayed various variation tendencies under different MTHF/H_2_O biphasic process conditions. Among these substrates, glucose yields of the R_170-60-0_ (33.6%) were similar to that of RM, implying that the MTHF/H_2_O process without adding H_2_SO_4_ did not have an effect on the reducing cornstalk materials recalcitrance. However, under the same MTHF/H_2_O temperatures and times (170 °C, 60 min) conditions, with the addition of H_2_SO_4_ from 0 to 0.05 M, the glucose yields appeared to notably increase from 33.6 to 78.9%. The increasing fact may be attributed to the enhancement of degradation and removal of hemicelluloses in RM during the MTHF/H_2_O system process under the current treatment conditions [30]. Nevertheless, as the H_2_SO_4_ concentrations further increased to 0.1 M, the glucose yields decreased to 65.0%, which was as a result of the degradation and removal of partial cellulose during the process. Under the same process times and H_2_SO_4_ concentrations (60 min, 0.1 M) conditions, the glucose yields obviously increased (59.4–73.9%) with the process temperatures increasing from 150 to 190 °C. Specifically, the effect of process times (30, 60, 120 min) under the same temperatures and H_2_SO_4_ concentrations (170 °C, 0.1 M) on glucose yields was indistinctive, and the overall values remained at 65.0–71.7%. It was noted that the resulting residue obtained from the maximum furfural preparation conditions (V_MTHF_:V_H2O_ = 1:1, 170 °C, 60 min, 0.05 M H_2_SO_4_) showed the highest glucose yield (78.9%). This was a very desirable result. In other words, it was important for the company to maximize both furfural and glucose yield under the same processing conditions.

### 3.4. Surface Morphology of the RM and Resulting Residues

**Figure 3:**
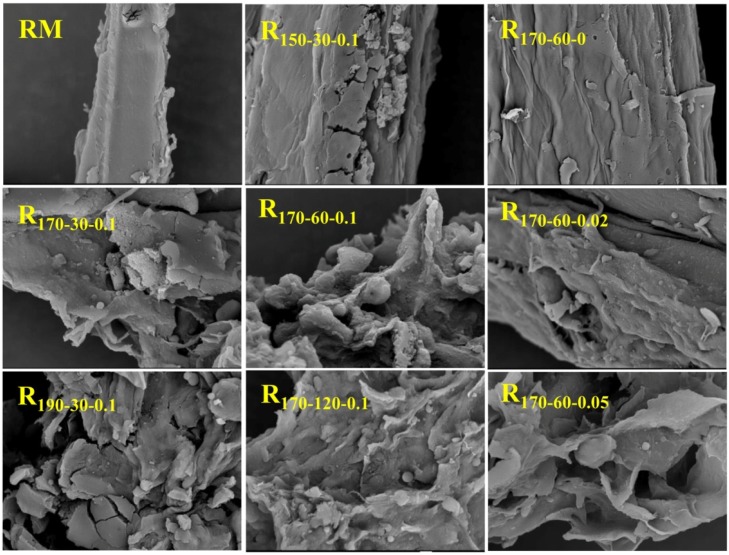
SEM images of the raw material and resulting solid residues with different systems.

To evaluate the various MTHF/H_2_O processes on the effect of surface morphology of RM, SEM images of resulting residues were observed at magnifications of 1000 (Figure 3). The surface of RM and R_170-60-0_ showed a smooth and dense morphology, resulting in enzymes having difficulty with contacting cellulose components in RM and R_170-60-0_ [31]. Thus, low glucose yields by the hydrolysis experiment were obtained from RM and R_170-60-0_. By contrast, the surfaces of the resulting residues obtained from the MTHF/H_2_O process by adding H_2_SO_4_ were broken and some cracks and particle-sized debris to different degrees emerged, which resulted in an increase of glucose yields of the residues [19]. For example, in all of the resulting residues obtained from the MTHF/H_2_O process under adding H_2_SO_4_ conditions, the surface damage degree of R_150-30-0.1_ was minimal, leading to the lowest glucose yield. As expected, as the H_2_SO_4_ concentrations increased from 0 to 0.05 M under the same process temperatures and times, the damage of the residues was aggravated. The fact indicated that the surface damage of the residues was conducive to the production of glucose by enzymatic hydrolysis. 

This phenomenon was mainly due to the increase in the accessibility of cellulase to cellulose by the release of a large number of adsorption sites [32]. When the H_2_SO_4_ concentration was further increased to 0.1 M, the surface of R_170-60-0.1_ was similar to R_170-60-0.05_. However, the glucose yields of two residues showed different values. This phenomenon suggested that the glucose yield was not only affected by surface morphology of the substrates, but also by several other factors, such as cellulose crystallinity and particle size of the substrates [33,34]. In short, the MTHF/H_2_O process led to surface destruction of RM, forming a large number of adsorption sites of cellulases on the surface of the substrates, thereby improving the efficiency of enzymatic hydrolysis.

### 3.5. FT-IR of the RM and Resulting Residues

**Figure 4:**
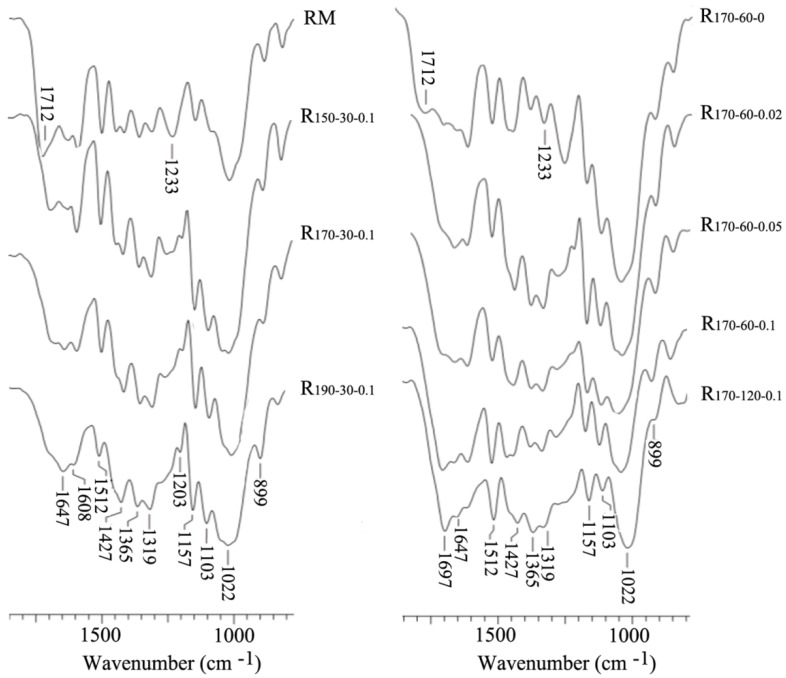
FT-IR spectra of the raw material and resulting solid residues with different systems.

To assess the structural changes of cornstalk after different MTHF/H_2_O processes, the FT-IR spectra of RM and resulting residues are illustrated in Figure 4. As can be seen, two obvious bands (1712 and 1233 cm^−1^) of the acetyl ester in hemicelluloses clearly appeared in the RM and R_170-60-0_ [35]. However, the two bands gradually weakened and even disappeared completely in the resulting residues under adding H_2_SO_4_ conditions, which was mainly significant removal of xylan hemicelluloses during the MTHF/H_2_O processes, especially at higher process severities. The presence of hemicelluloses inhibited the enzymatic hydrolysis efficiency of cellulose in different biomasses to some extent [36]. Therefore, the removal of hemicelluloses was beneficial to improve the glucose yield, and the results of enzymatic hydrolysis also confirmed this conclusion. The bands about aromatic skeletal vibrations and the C−H deformation were distinctly observed at 1608, 1512, and 1427 cm^−1^ in all the spectra of the resulting residues [37,38]. This suggested that the enhancement of the process severities during the MTHF/H_2_O system had no evident influence on the structure and content of the lignin. Thus, after the MTHF/H_2_O processes, the improvement of the enzymatic hydrolysis efficiency of the resulting residues was mainly attributed to the removal of hemicellulosic constituents from the RM.

### 3.6. Crystallinity of Cellulose in the RM and Resulting Solid Residues

**Figure 5:**
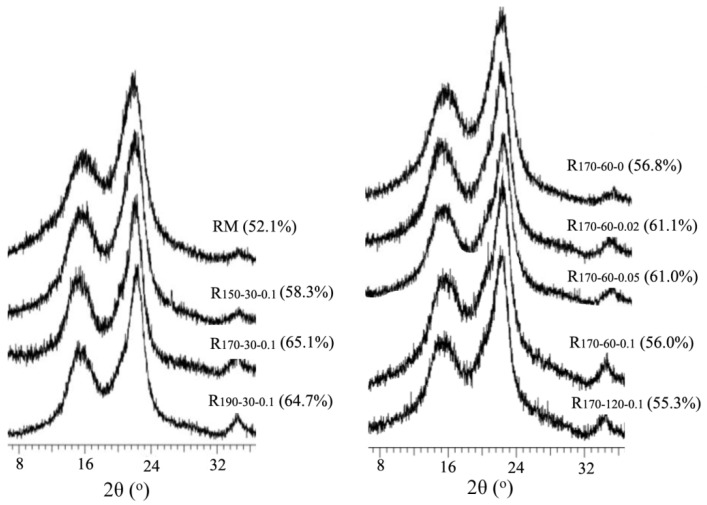
XRD spectra of the raw material and resulting solid residues with different systems.

Besides the contents and chemical structures of hemicelluloses and lignin as well as surface morphology of the substrates affecting the glucose yields of resulting residues, the CrI is also considered one of the important factors influencing the production of glucose [39,40]. Thus, the CrIs of cornstalk before and after the various MTHF/H_2_O processes were determined by XRD patterns (Figure 5). As can be seen, the crystal structure of cellulose in resulting residues collected obtained from the different MTHF/H_2_O processes did not transform, but there was increasing evidence in the CrIs (55.3–65.1%) compared to that of RM (52.1%). Among these residues, the CrI of the R_170-60-0_ was only increased by 4.7%, so the glucose yields of the RM and R_170-60-0_ showed similar values. As the elevating process severities, the CrIs of the resulting residues first increased and then decreased, implying that the partial cellulose occurred degradation under the higher severities [41]. Combined with the results of glucose yields of the substrates, it was found that there was no linear relation between CrIs and glucose yields of resulting residues since the hydrolysis efficiency of cellulose was not only affected by CrI, but also by several other factors, such as contents and distribution of hemicelluloses or lignins as well as surface morphology of the substrates [42,43,44,45,46]. Cheng et al. examined the influence of the ionic liquid process on the crystal structure of cellulose in different biomasses (microcrystalline cellulose, switchgrass, pine, and eucalyptus), and its effect on hydrolysis kinetics of cellulose. The results indicated that the biphasic process led to a loss of crystalline region for native cellulose. Particularly, there was a significant difference in the transformation process between microcrystalline cellulose and lignocellulosic samples. Microcrystalline cellulose was explained by more thoroughly transforming to cellulose II after the process under the condition given. However, the lignocellulosic samples showed that another other factor, likely lignin-carbohydrate complexes, also impacted the hydrolysis efficiency in addition to CrI [46]. Thus, a single factor may not be sufficient to detail the difference in glucose production, and several factors affected the cellulose hydrolysis of the substrate in the current study [47].

## 4. Conclusions

Simultaneous and efficient production of furfural and subsequent glucose in the MTHF/H_2_O biphasic system via parameter regulation was a promising approach to convert cornstalk. The results demonstrated that the MTHF/H_2_O conditions (V_MTHF_/V_H2O_, temperature, time, and H_2_SO_4_ concentration) had a remarkable influence on the furfural and glucose preparation. The maximum furfural yield was 68.1% under the reaction conditions (V_MTHF_:V_H2O_ = 1:1, 170 °C, 60 min, 0.05 M H_2_SO_4_). The concentration of H_2_SO_4_ was the most important parameter for furfural production. The glucose yields of cellulose were improved after different MTHF/H_2_O processes and a maximum value was up to 78.9% under the same MTHF/H_2_O system condition with preparation of furfural. The single factor may not adequately elaborate the differences of glucose yield, and several factors impacted the cellulose hydrolysis of the substrates.

## Figures and Tables

**Table 1:** Yields (%, w/w) and chemical compositions (%, w/w) of the resulting solid residue obtained by different 2-Methyltetrahydrofuran/aqueous (MTHF/H_2_O) systems.

Entry	Cellulose ^a^	Hemicelluloses	KL	Yields
R_170-60-0_	54.7	7.6	21.6	59.0
R_170-60-0.02_	62.8	ND ^b^	23.0	57.9
R_170-60-0.05_	64.3	ND	27.0	50.1
R_170-60-0.1_	51.1	ND	28.6	48.9
R_150-30-0.1_	65.4	ND	23.2	50.0
R_170-30-0.1_	67.1	ND	24.0	49.7

^a^ Cellulose was expressed as glucan and hemicelluloses was expressed as xylan, arabinan, and galactan. KL was Klason lignin (i.e., acid insoluble lignin) while ASL was acid soluble lignin. ^b^ ND, not detected.
